# Next-Generation Sequencing of Genomic DNA Fragments Bound to a Transcription Factor *in Vitro* Reveals Its Regulatory Potential

**DOI:** 10.3390/genes5041115

**Published:** 2014-12-19

**Authors:** Yukio Kurihara, Yuko Makita, Mika Kawashima, Hidefumi Hamasaki, Yoshiharu Y. Yamamoto, Minami Matsui

**Affiliations:** 1 Synthetic Genomics Research Team, Biomass Engineering Program Cooperation Division, RIKEN Center for Sustainable Resource Science, 1-7-22 Suehiro-cho, Tsurumi-ku, Yokohama, Kanagawa, 230-0045, Japan; E-Mails: yukio.kurihara@riken.jp (Y.K.); yuko.makita@riken.jp (Y.M.); mkawa@riken.jp (M.K.); hamasaki@psc.riken.jp (H.H.); 2 The United Graduate School of Agricultural Science, Faculty of Applied Biological Sciences, Gifu University, 1-1 Yanagido, Gifu, 501-1193, Japan; E-Mail: yyy@gifu-u.ac.jp

**Keywords:** plant, transcription factor, HY5, *in vitro* binding, next-generation sequencing

## Abstract

Several transcription factors (TFs) coordinate to regulate expression of specific genes at the transcriptional level. In *Arabidopsis thaliana* it is estimated that approximately 10% of all genes encode TFs or TF-like proteins. It is important to identify target genes that are directly regulated by TFs in order to understand the complete picture of a plant’s transcriptome profile. Here, we investigate the role of the LONG HYPOCOTYL5 (HY5) transcription factor that acts as a regulator of photomorphogenesis. We used an *in vitro* genomic DNA binding assay coupled with immunoprecipitation and next-generation sequencing (gDB-seq) instead of the *in vivo* chromatin immunoprecipitation (ChIP)-based methods. The results demonstrate that the HY5-binding motif predicted here was similar to the motif reported previously and that *in vitro* HY5-binding loci largely overlapped with the HY5-targeted candidate genes identified in previous ChIP-chip analysis. By combining these results with microarray analysis, we identified hundreds of HY5-binding genes that were differentially expressed in *hy5*. We also observed delayed induction of some transcripts of HY5-binding genes in *hy5* mutants in response to blue-light exposure after dark treatment. Thus, an *in vitro* gDNA-binding assay coupled with sequencing is a convenient and powerful method to bridge the gap between identifying TF binding potential and establishing function.

## 1. Introduction

Changes in gene expression profiles induced by environmental stimuli or developmental phase shifts are controlled through specific multiple mechanisms at the transcriptional, post-transcriptional and translational levels. Transcription is positively or negatively controlled by several transcription factors (TFs) [1,2]. TFs with DNA binding potential generally bind to the genic region, such as the promoter or enhancer regions of a gene to activate or inactivate its expression. In *Arabidopsis thaliana* it is estimated that approximately 10% (~3000) of all genes encode TF or TF-like proteins based on the TAIR (The Arabidopsis Information Resource) annotation. TFs often play central roles in developmental phases during the formation of organs and tissues. Moreover, in response to environmental changes, several TFs coordinate to regulate the expressional switch of specific genes and enable the organism to adapt to its modified surroundings. For example, APETALA2 TF guides proper flower and fruit development [3,4], while the DREB and AREB family TFs regulate expression of the downstream genes involved in drought stress and abscisic acid responses, respectively [5,6,7,8].

Since these TFs may form complicated networks in order to execute their function, it is important to identify the target genes that are regulated by TFs in order to understand the environmental response of the whole plant. Chromatin immunoprecipitation (ChIP) followed by microarray hybridization (ChIP-chip) or next-generation sequencing (ChIP-seq) has generally been used to identify genes targeted by TFs [9,10,11,12]. Whilst these methods are powerful, they require sophisticated skills and, in many cases, well-purified specific antibodies against the TFs of interest. In addition, transgenic plants expressing the TFs, which are time-consuming to generate, are sometimes required for the ChIP assay. Previous work established a rapid method for identifying targets of a DNA-binding protein, named DIP-chip, in which purified proteins and sheared gDNA fragments are mixed *in vitro*. Protein-gDNAs are immunoprecipitated and then gDNA that was bound to the proteins is hybridized on a tiling array [13]. This method reveals *in vivo* binding motifs and genomic positions.

LONG HYPOCOTYL5 (HY5) is a bZIP-type transcription factor that extensively regulates photomorphogenesis through binding to light-inducible or light-repressed genes in plants [14,15,16]. HY5 acts downstream of photoreceptors, phytochromes and cryptochromes [17]. The loss-of-function *Arabidopsis* mutant of the *HY5* gene is insensitive to light and shows a long hypocotyl phenotype under different lights including red, far-red and blue lights [18]. Previous ChIP-chip analysis identified more than 3000 HY5 binding sites in the genome [11]. In addition, another report demonstrated by *in vitro* binding assays that HY5 recognizes ACGT-containing sequence motifs [19].

TFs for *in vitro* DNA binding assays are often produced using a protein synthesis system in *E. coli* but this system requires different codon usage from eukaryotes and gives no post-translational modifications. Wheat germ extract is a powerful cell-free tool to synthesize large amounts of eukaryotic proteins from *in vitro* transcribed mRNAs [20,21]. Proteins synthesized by this method possess post-translational modifications. In order to understand the complete transcriptional network orchestrated by TFs, it is important to establish a more convenient method than ChIP-chip, ChIP-seq or DIP-chip. In this report, we first established an *in vitro* gDNA binding assay like the DIP method using HY5 protein produced by the wheat germ extract system as a model TF and performed next-generation sequencing to identify TF-binding regions. We also show the relationship between the binding targets and the blue light response revealed by RNA-seq. Here, we demonstrate a powerful use of an established method to reveal the gDNA binding potential of TFs.

## 2. Experimental Section

### 2.1. Protein Synthesis

The coding sequence of HY5 (AT5G11260) was amplified from a cDNA clone by PCR using PrimeSTAR max polymerase (Takara) and two primers, 5'-ACTCGAGATGCAGGAACAAGCGACTAGCTC-3' and 5'-AGCGGCCGCTCAAGCATAATCTGGTACATCATATGGATAAAGGCTTGCATCAGCATTAGAAC-3', the latter of which includes a sequence for a HA tag, and subcloned into the pCRII-TOPO vector (Life Technologies) creating pCRII-HY5-HA. The HY5-HA fragment arising from restriction cleavage of pCRII-HY5-HA with XhoI and NotI was inserted into equivalent sites of the pEU vector (Cell Free Science) creating pEU-His-HY5-HA. For protein synthesis from pEU-His-HY5-HA, *in vitro* transcription and translation in wheat germ extract (WEPRO7240H Expression kit) using the Protemist-DT II, an automatic machine (Cell Free Science), was performed according to manufacturer’s instructions. Protein synthesis was performed in a 6 mL volume. After purification of the proteins the solvent (20 mM sodium phosphate pH 7.5, 0.3 M NaCl, 0.5 M imidazole) was replaced with storage buffer (1× phosphate buffered saline (PBS; 137 mM NaCl, 2.7 mM KCl, 10 mM Na_2_HPO_4_, 1.76 mM KH_2_PO_4_) pH 7.4, 10% glycerol, 5 mM EDTA) using Amicon Ultra Centrifugal Filters (Ultracel-10K) (Millipore) to a final volume of 250 µL.

### 2.2. SDS-PAGE and Western Blot

The synthesized proteins were loaded onto a 12% SDS-PAGE gel for electrophoresis and then stained with CBB for visualization. Alternatively, after electrophoresis, proteins were transferred onto an Immobilon-P membrane (Millipore) followed by blocking in buffer (1× Tris-buffered saline (TBS; 25 mM Tris-HCI pH 7.4, 137 mM NaCl, 2.7 mM KCl), 3% skimmed milk). Anti-HA antibody conjugated with peroxidase (Roche), anti-HY5 antiserum and anti-rabbit antibody with peroxidase (GE Healthcare) were used for detection of HY5-HA. Proteins were visualized by the ECL Prime Western Blotting Detection System and detected using an ImageQuant LAS 4000 (GE Healthcare).

### 2.3. DNA Immunoprecipitation, Sequencing and Analysis

Genomic DNA (gDNA) was extracted from the whole aerial portion of two-to-four-week-old Arabidopsis Col-0 plants using a DNeasy Plant Midi Kit (Qiagen) and sheared into lengths of approximately 150–200 nt by Covaris S (Covaris).

Approximately 10 µg of recombinant HY5-HA protein were mixed with a 60 µL volume of Dynabeads Protein G (VERITAS) and 5 µg of anti-HA antibody (abcam) in 400 µL of solution A (1× PBS pH 7.4, 5 mM EDTA, 0.05% Triton X-100, 5% glycerol) and then incubated with constant rotation for two hours at room temperature. The beads combined with HY5-HA were washed twice using solution A and resuspended in 500 µL of solution A. The sheared gDNA fragments (25 µg) were added into the solution containing the beads with HY5-HA and incubated with constant rotation overnight at 4 °C. The HY5-HA beads bound with gDNA fragments were washed four times in solution A and resuspended in 100 µL of solution B (10 mM Tris-HCl pH 8.0, 10 mM EDTA, 0.5% SDS). This was mixed with 5 µL of proteinase K (2 mg/mL) and incubated for two hours at 37 °C. The gDNA fragments were recovered by phenol:chloroform, and chloroform extractions followed by ethanol precipitation. A library for next-generation sequencing was constructed using the TruSeq ChIP Sample Prep Kit (Illumina) and the pair-end sequencing (2 × 150 bp) was performed using a MiSeq sequencer (Illumina) according to the manufacturer’s instructions.

The reads (approx. 2 × 12.6 M reads) obtained as fastq files were mapped onto the genome by the Bowtie 2 program [22] and peak detection (*q*-value < 0.01) of gDB-seq was performed using the MACS2 program [23]. Approximately 25.3 M reads were mapped. Peak scores were defined as −10 × LOG10 (*p*-value) in MACS2. Using the GADEM program [24], we detected candidate motifs and the JASPAR database was used to carry out a motif similarity search [25].

Determination of distance from TSS to HY5-binding sites (Figure 2B) was done based on information of “Genic Top TSS Peak” that represents one peak for one gene [26].

### 2.4. Microarray Analysis

Seeds were sown on filter paper that was put onto medium (1× Murashige Skoog pH 5.8, 0.8% agar) and incubated under white light for three days. Total RNAs were extracted using a RNeasy Plant Mini Kit (Qiagen). Labeling of the RNAs with Cy3 was performed by a One-color Quick Amp Labeling Kit (Agilent). Hybridization to Agilent Arabidopsis 44K microarray was performed using a Gene Expression Hybridization Kit in an oven according to the manufacturer’s instructions (Agilent). Signals were obtained using a DNA Microarray Scanner (Agilent). Three replicates of the microarray analyses were performed in wild type and *hy5* (SALK_096651) [27]. For data analysis, signals of the raw data were normalized to each value of the third quartile point. Significant differences were defined by *Welch*’s *t*-test (*p* < 0.05) and 2- or 0.5-fold changes.

### 2.5. RNA-Seq Analysis

Three-day-old wild-type Col-0 plants grown on filter paper in the dark, as described above, were exposed to blue light and harvested after one-hour exposure. Total RNAs were extracted using Trizol reagent (Life Technologies) and purified by PureLink RNA Mini Kits (Life Technologies). Directional RNA-seq libraries were constructed using TruSeq Small RNA Sample Prep Kits and TruSeq RNA Sample Preparation Kits according to the Directional mRNA-Seq Library Prep. Manual (Illumina) and sequenced using a HiSeq sequencer (Illumina). The reads (60 nt, fastq files) were mapped onto the genomic sequence by the Tophat program (max_segment_intron, 500; library_type, fr-unstranded) [28] and FPKM values were calculated using the Cufflinks program [29]. Significant difference was defined by three values, *Welch*’s *t*-test (*p* < 0.05), FPKM average > 1 and 2- or 0.5-fold changes.

### 2.6. Semi-Quantitative RT-PCR Analysis

Total RNAs were extracted using the RNeasy Plant Mini Kit (Qiagen) and then digested with DNase I (Takara). Reverse transcription from 1 µg of the total RNA was performed using the PrimeScript II first strand cDNA Synthesis Kit (Takara). Quantitative PCRs were performed using PrimeSTAR max polymerase (Takara). Primers used here are listed in Table S1. PCR products were loaded onto a 1.5% TBE agarose gel containing SYBR Gold Nucleic Acid Gel Stain (Life Technologies) and visualized under ultra-violet light.

## 3. Results and Discussion

### 3.1. Synthesis of Recombinant HY5 Protein

**Figure 1:**
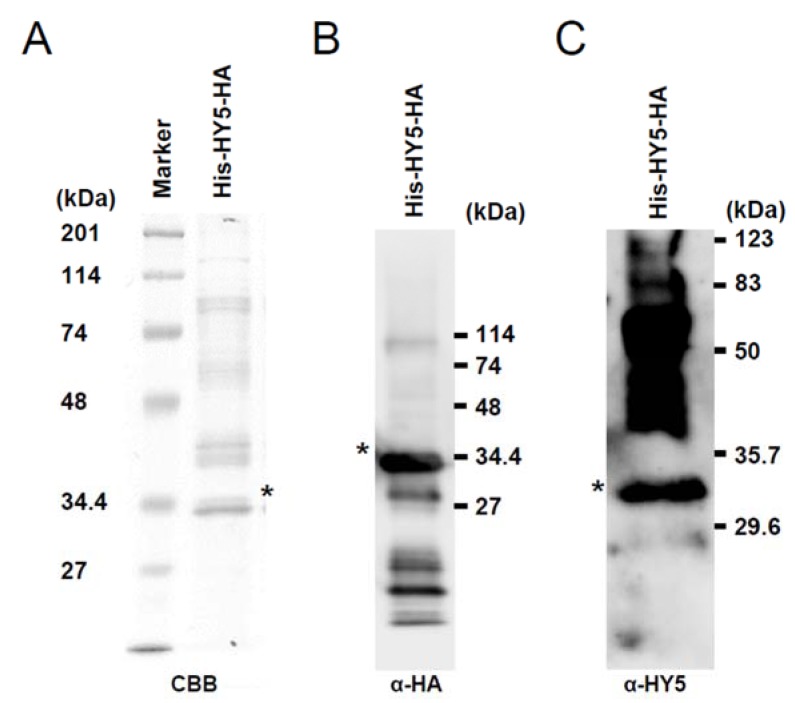
Recombinant His-HY5-HA protein produced in wheat embryo extract. (**A**) CBB (Coomassie Brilliant Blue) staining of the gel on which purified proteins were loaded. (**B**) and (**C**) Western blot analysis of the purified proteins using anti-HA antibody (**B**) and anti-HY5 antiserum (**C**), respectively. Asterisks indicate the positions of His-HY5-HA, respectively.

A cell-free expression system in wheat germ extract was used to produce recombinant HY5 proteins with a His-tag at the N terminal and a HA-tag at the C terminal (His-HY5-HA). Purified recombinant proteins were subjected to gel electrophoresis and visualized by Coomassie Brilliant Blue (CBB) staining (Figure 1A). Production of HY5 protein was also confirmed by Western blot analysis using anti-HA antibody (Figure 1B, asterisk) and anti-HY5 antibody (Figure 1C, asterisk). The molecular weight of the detected protein was approximately 34 kDa, whilst the predicted molecular weight was 20.8 kDa. A similar difference between *in vivo* synthesized HY5 and its predicted molecular weight has been reported previously [15]. This difference indicates that the HY5 protein produced in the wheat germ extract has undergone unknown post-translational modifications. Thus, we could use a form of recombinant HY5 protein that was similar to the *in vivo* form for the following experiments.

### 3.2. Establishment of *in Vitro* Genomic DNA Binding Assay

**Figure 2:**
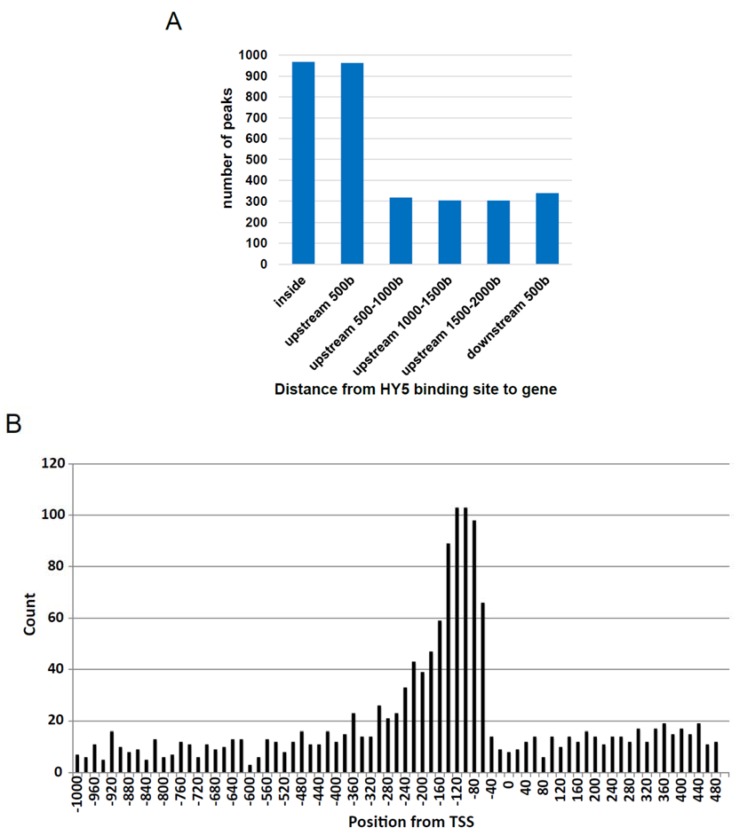
Peak positions derived from HY5 gDB-seq. (**A**) Positions of detected peaks around the HY5-binding genes. (**B**) Distribution of gDB-seq peak positions from transcription start sites (TSSs).

We established a method to identify HY5 genomic binding sites based on *in vitro* gDNA immunoprecipitation like DIP-chip [13] and next-generation sequencing. A detailed description is given in the Materials and Methods section. Firstly, recombinant HY5 proteins, His-HY5-HA, were bound to beads, mixed with sheared gDNA fragments (150–200 bp) and incubated *in vitro*. After immunoprecipitation using anti-HA antibodies, gDNA fragments bound with His-HY5-HA were recovered and subjected to next-generation sequencing. The read sequences were mapped onto the *Arabidopsis* genome and then peak binding locations were detected *in silico*. We detected 2503 HY5-binding peaks located in the *Arabidopsis* genome and 17 peaks located to the mitochondrial or chloroplast genomes (MACS2 *q*-value < 0.01). Of the nuclear genome peaks, 2177 mapped to the regulatory regions of 3195 nuclear genes. These regions are defined as being from 2000 bases upstream to 500 bases downstream of the gene (Figure 2A and Table S2). Of these, 60% are located either 500 bases upstream (the promoter) or inside the genes (gene bodies), although some peak positions mapped 500 bases downstream. In addition, we compared the HY5-binding peak positions with TSSs (transcription start sites) that have been identified previously [26]. Many of the positions were strongly associated with the 200-base (promoter) region upstream from the TSSs (Figure 2B). On the other hand, 326 peaks mapped to intergenic regions. We named this method that identifies TF-binding sites from genomic fragments as the gDNA binding sequencing method (gDB-seq).

**Figure 3:**
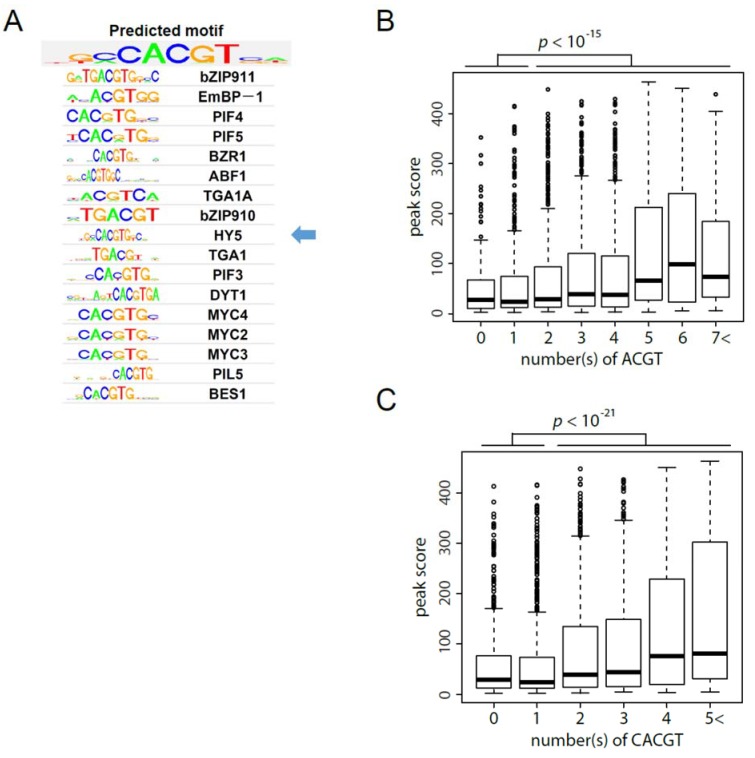
Motif prediction of HY5-binding sequence. (**A**) Results of the HY5-binding motif prediction and similarity search in the JASPAR database. Seventeen TFs with similar motifs as those of HY5 are shown under the predicted motif. Arrow indicates a HY5 binding motif registered in the JASPAR database. (**B**) and (**C**) box plots of the distribution of peak scores to number(s) of ACGT (**B**) and CACGT (**C**) sequence(s), respectively. *p*-values of peak scores between categories (0 and 1) and (2<) are shown above boxplots. Peak numbers included in each category of (**B**) and (**C**) are 219 (0), 498 (1), 599 (2), 562 (3), 378 (4), 163 (5), 68 (6) and 33 (7<), and 544 (0), 809 (1), 659 (2), 344 (3), 113 (4) and 51 (5<), respectively.

We predicted five conserved sequence motifs of the HY5-binding sites using the surrounding sequences of 498 peaks with the highest peak scores using GADEM program [24] and found that one of the five conserved sequences contains 5'-(G/T)(C/A)CACGT(C/G)-3' that is similar to the HY5-binding motif registered in the JASPAR database, indicating that the motif prediction is almost accurate (Figure 3A and Figure S1) [25]. Of the 2520 detected peaks, 2301 and 1976 peaks contain at least one ACGT or CACGT consensus sequence(s), respectively, within 400 bases of the peaks (Table S3). The number of ACGT- or CACGT-containing sequences in the gene-related regions tended to correlate with the peak scores (Figure 3B and Figure 3C). These observations indicated that the gDB-seq of HY5 sufficiently uncovers binding motif and genomic binding sites.

**Figure 4:**
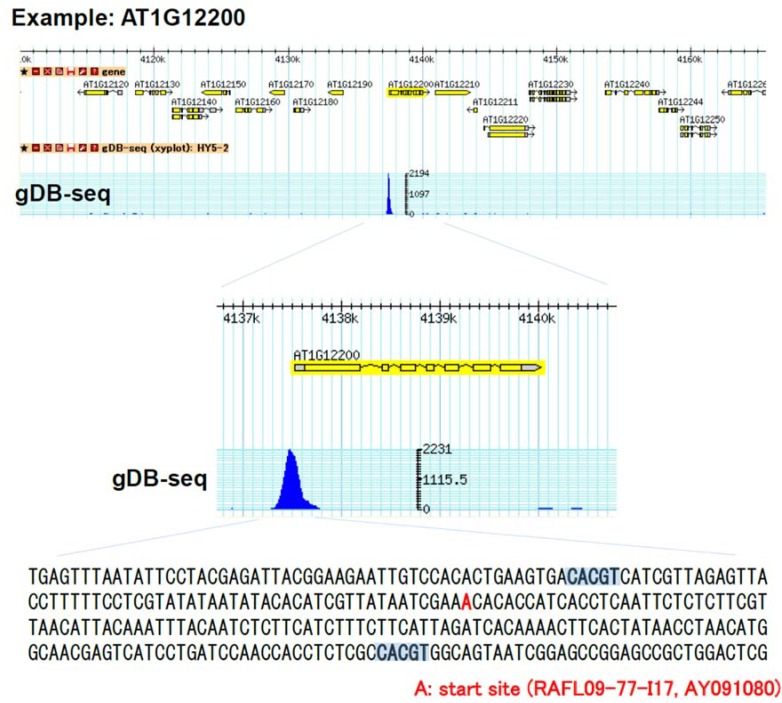
Genome browser view of an example of gDB-seq. There is one peak at the promoter of the AT1G12200 gene. Sequence around this peak contains two predicted motifs (CACGT). Peak score = 218.0. The HY5 gDB-seq data can be visualized using GBrowse located at http://plant.psc.riken.jp/cgi-bin/gb2/gbrowse/arabidopsis.

The HY5 gDB-seq data can be visualized using GBrowse located at http://plant.psc.riken.jp/cgi-bin/gb2/gbrowse/arabidopsis. For example, in the promoter region of *AT1G12200*, there is a peak in which there are two CACGT-containing motif sequences (Figure 4). In ChIP-based methods, several factors in the nucleus may affect the selection of binding sites by the TF and the TF’s binding affinity. On the other hand, a uniform pool of gDNA fragments was mixed with recombinant TF proteins thus avoiding any involvement of other factors in the binding assay of gDB-seq. Therefore, using gDB-seq it is possible to find the relative TF affinity (peak score) to a binding site compared with those of other binding sites. For example, the score (218.0) of the peak located upstream of AT1G12200 (Figure 4) is higher than that (128.6) of the peak located upstream of AT1G10090, indicating that HY5 binds upstream of AT1G12200 more strongly than it does upstream of AT1G10090 (Table S2).

Some studies have established novel *in vitro* high-throughput methodologies for identifying TF-binding motifs, such as SELEX (systematic evolution of ligands by experimental enrichment)-seq and PBM (protein-binding microarray) methods [30,31,32,33]. Compared with these methods, gDB-seq and ChIP-based methods are good at identifying candidate genes targeted by the TFs as well as binding motifs. In addition, it is possible that gDB-seq uncovers larger number of possible physical binding sites of a TF than ChIP-based methods, because gDB-seq eliminates some *in vivo* interferences, such as environmental conditions, cell types and interaction with other cellular factors.

### 3.3. Comparison of gDB-Seq with ChIP-Chip

**Figure 5:**
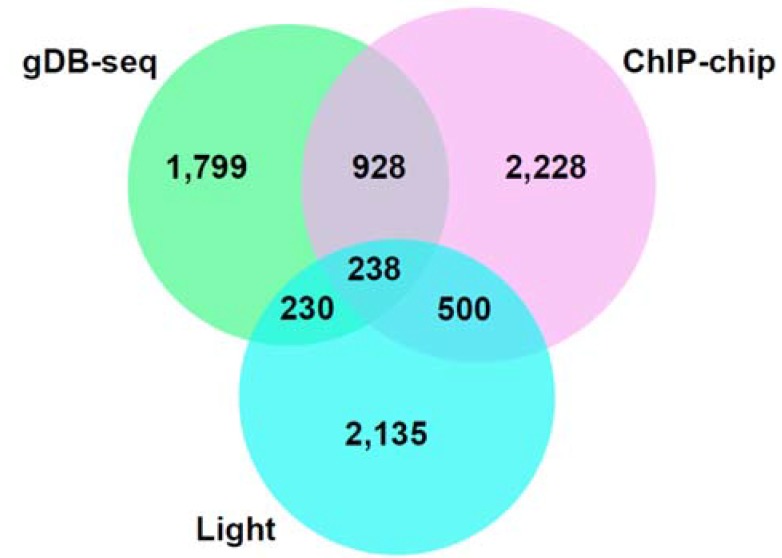
Venn diagram of overlaps between gDB-seq (gDNA binding sequencing), ChIP-chip (Chromatin immunoprecipitation-chip) [11] and light-regulated genes [34].

In previous ChIP-chip work, 3894 genes were estimated to be binding targets of HY5 [11] and 738 of them overlapped with 3103 light-regulated genes reported previously by microarray analysis using RNAs of cotyledon, hypocotyl and root [34]. To evaluate the gDB-seq results we compared 3195 HY5-binding candidate genes identified through the gDB-seq method with those of the *in vivo* ChIP-chip analysis and also with light-regulated genes. About 36.5% (1166) or 14.6% (468) of candidate genes in gDB-seq overlapped with genes in ChIP-chip or light-regulated genes, respectively (Figure 5 and Table S2).

**Figure 6:**
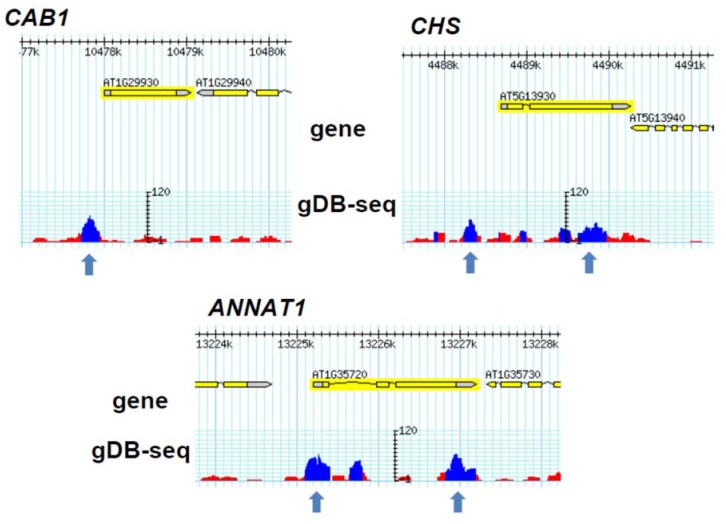
Genome browser view of the *CAB1*, *CHS* and *ANNAT1* loci. Arrows indicate possible peak positions, which were not detected in MACS2 (Model-based Analysis of ChIP-Seq) peak prediction.

Lee *et al*. selectively picked up and analyzed four genes, *CAB1*, *CHS*, *RbcS1A* and *F3H*, which were detected by ChIP-chip of HY5 [11]. While the *F3H* and *RbcS1A* genes were also predicted to be HY5-binding genes in this report, the *CAB1* and *CHS* genes were not. When we examined their loci on the genome browser, we observed much lower peaks comprising of small numbers of sequenced reads on both the *CAB1* and *CHS* loci rather than the predicted peaks (Figure 6). The *ANNAT1* gene was also detected by ChIP-chip of HY5 [11], but not by gDB-seq. However, using a cut-off with a higher *q*-value (<0.05) in the MACS2 program resulted in the detection of a peak located in the *ANNAT1* gene (Figure 6), suggesting that the number of gDB-seq peaks may increase if we used different ways to predict the peak positions. Alternatively, as the DNA-protein binding buffer condition could affect gDB-seq results, any changes in the buffer may lead to different results from this report.

### 3.4. Association Study between gDB-Seq and Microarray in hy5 Mutant

**Figure 7:**
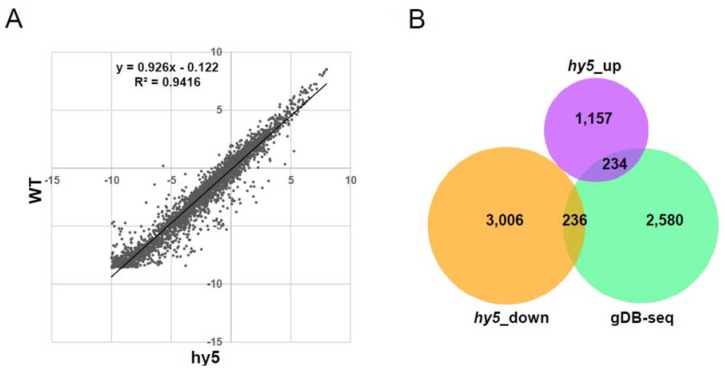
Microarray analysis in *hy5* null mutant. (**A**) Dot plot of the accumulation of 3050 HY5-binding genes in wild type (WT) and *hy5*. LOG2 values of normalized signals were plotted. (**B**) Venn diagram of overlaps between HY5-binding genes and genes that showed a significant difference in microarray analysis in the *hy5* mutant.

To identify direct targets regulated by HY5 binding, we performed microarray analysis of three-day-old *hy5* null mutants grown under continuous white light. We found that 1391 or 3242 genes were up-regulated or down-regulated in *hy5* mutants, respectively, compared to wild type (twofold and *p*-value < 0.05). In this microarray, probes of 3050 genes of the 3195 HY5-binding genes were plotted and accumulation of most of their transcripts in the *hy5* mutants was equivalent to those in wild-type plants (Figure 7A). However, 234 or 236 transcripts of 3050 HY5-binding genes overlapped with the up-regulated or down-regulated transcripts in the *hy5* mutants, respectively (Figure 7B and Table S4). These include novel HY5-regulated candidate genes that were not identified previously [11]. These results indicate that HY5-binding potential does not necessarily associate with the accumulation of transcripts from the HY5-targeted genes and that secondary effects by disruption of the *HY5* gene had a much larger impact on the whole transcriptome than any direct effect.

### 3.5. Role of HY5-Binding Potential in Response to Blue Light Exposure

**Figure 8:**
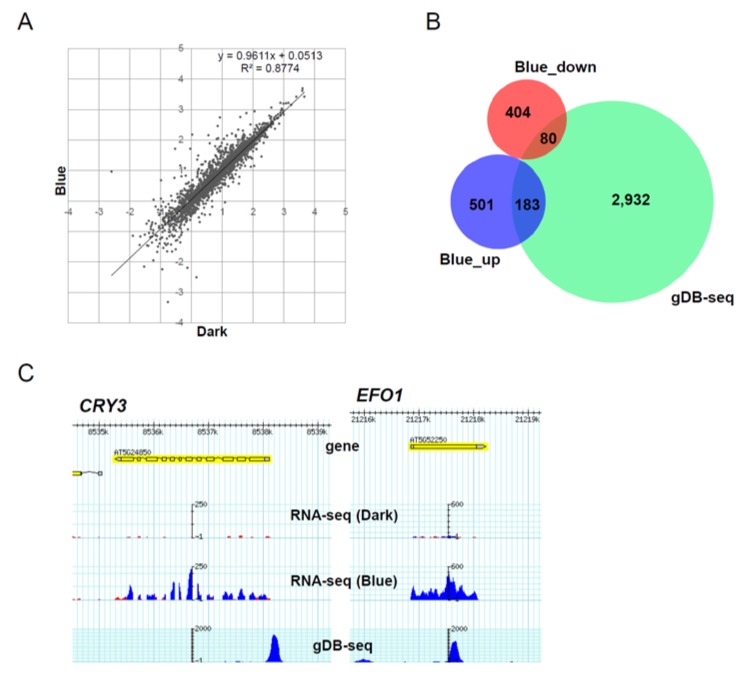

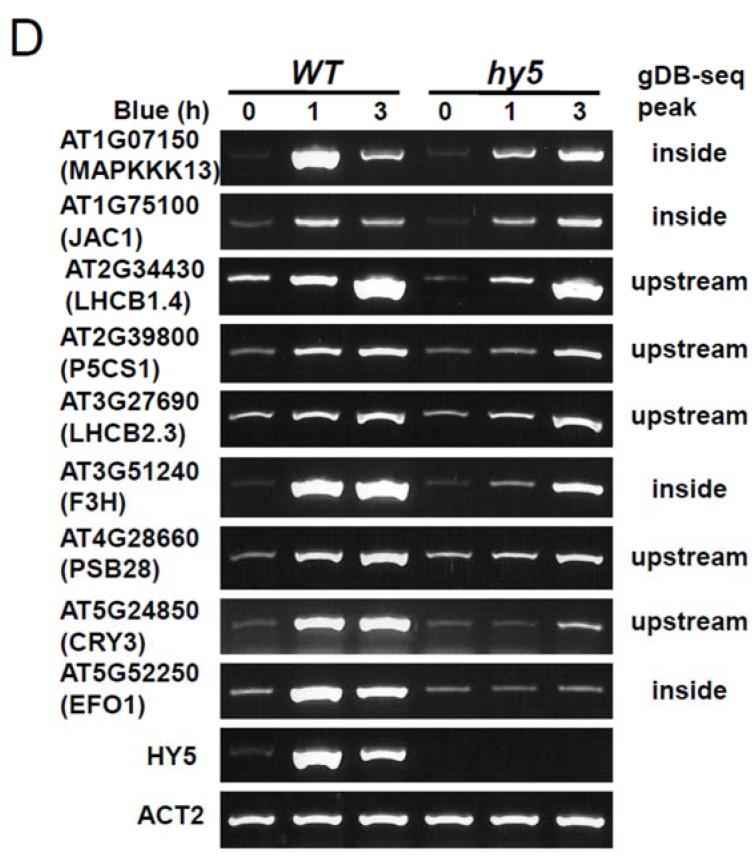
Relationship between HY-5 binding potential and early blue-light response. (**A**) Dot plot of the accumulation of 2631 HY5-binding genes in plants grown in the dark and under blue light for 1 hour. LOG10 (in −4~4) values of FPKMs (Fragments per kilobase of exon per million mapped reads) were plotted. (**B**) Venn diagram of the overlaps between HY5-binding genes (gDB-seq), blue-inducible genes (Blue_up) and blue-repressed genes (Blue_down). (**C**) Genome browser view of two examples (the *CRY3* and *EFO1* loci) of blue light-inducible genes with HY5-binding sites. (**D**) Semi-quantitative RT-PCR (Reverse Transcription-Polymerase Chain Reaction) analysis of nine HY5-binding genes, *HY5* and *ACT2*. *ACT2* accumulation was used as a loading control. The peak positions of gDB-seq are shown on the right.

As shown in Figure 5, several HY5-binding genes overlapped with light-regulated genes. In order to know the transcript profiles of the HY5-binding genes in response to blue light exposure, three-day-old plants grown in the dark were transferred to blue light, grown for one hour under blue light, and harvested. We then performed directional RNA-seq analysis and the results showed that 684 or 484 transcripts were up-regulated or down-regulated, respectively. The accumulation tendency of the transcripts from HY5-binding genes is comparable between dark and blue light (Figure 8A). Nevertheless, 80 down-regulated and 183 up-regulated genes in *hy5* overlapped with HY5-binding genes (Figure 8B and Table S5). For example, blue-light inducible genes, *CRY3* and *EFO1*, possess gDB-seq peaks at the promoter and gene body, respectively (Figure 8C).

To understand the role of HY5 binding on blue-inducible genes, we examined the change in accumulation of the transcripts of nine blue-inducible HY5-binding genes by semi-quantitative RT-PCR analysis. We detected delayed induction of eight transcripts including *MAPKKK13*, *JAC1* and *F3H* in *hy5* mutants compared with wild type (Figure 8D). This result suggests that HY5 binding positively regulates efficient induction of blue-light inducible genes *in vivo*. Interestingly, it is possible that HY5 binds and affects gene expression not only by binding promoters but also by binding gene bodies, such as the *EFO1* locus (Figure 8C and Figure 8D).

The result above is one example of how gDB-seq analysis will contribute to future research that will clarify the regulatory mode of transcription by TFs during environmental change in plants. Generally, *in vivo* ChIP-based methods identify physiological TF-binding sites, while *in vitro* gDB-seq, reveals the candidate genes regulated by a TF. The information from gDB-seq will be useful to elucidate the whole picture of genes that are controlled by TFs.

HY5 regulates blue-light signaling pathway through physical interaction with other bZIP-type TFs, HYH and GBF1 [18,35]. GBF1 antagonistically acts with HY5 and HYH in seedling development [18]. HY5 also interacts with some B-Box-containing TFs like BBX21, BBX22 and BBX25 [36,37,38]. While BBX21 and BBX22 act as positive regulators of photomorphogenesis [36,37], BBX25 does as a negative regulator by down-regulating *BBX22* expression [38]. The output of gDB-seq ignores these interactions and chromatin states, which are often important for *in vivo* regulation of gene expression by HY5. Therefore, additional studies through other approaches may be required to reveal regulation of genes targeted by HY5 based on gDB-seq information.

## 4. Conclusions

In this research, we established an *in vitro* methodology for identifying TF-binding sites based on protein synthesis in wheat germ extract, DNA immunoprecipitation and next-generation sequencing. This method was applied to the HY5 transcription factor, a key regulator of photomorphogenesis. Comparison between our gDB-seq and previous ChIP-chip analyses suggests that *in vitro* gDB-seq covers the *in vivo* binding sites of HY5 to some extent. *In vivo* ChIP-chip and ChIP-seq reveal physiological binding sites of TFs, while *in vitro* gDB-seq identifies a comprehensive set of physical binding sites. Therefore, this method will be useful to understand the overall nature of physical TF binding sites and identify their affinity strengths and conserved motifs.

## Supplementary Files

Supplementary File 1

Supplementary File 2

## Data Deposit

Arrays and sequences have been submitted to the GEO and SRA at NCBI as GSE62119, SRP048725 and SRP048726.
