# Supplementary material for: Next-Generation Sequencing of Genomic DNA Fragments Bound to a Transcription Factor in Vitro Reveals Its Regulatory Potential
Source: Genes (Basel). 2014 Dec 19;5(4):1115–31. doi: 10.3390/genes5041115 (PMC4276929; doi:10.3390/genes5041115)
Supplement: Supplementary File 2 [file genes-05-01115-s002.docx]

**Supplementary Materials**

**Figure S1.** Five predicted sequence motifs of HY5-binding sites using the surrounding sequences of 498 peaks with the highest scores. Twenty TFs with similar motifs as those of HY5 are shown under the predicted motif (top).
